# ALPACA: a visual data mining system for subcellular location-specific knowledge mining from multi-omics data in cancer

**DOI:** 10.1186/s12920-026-02440-4

**Published:** 2026-11-06

**Authors:** Minwoo Pak, Dabin Jeong, Sungjoon Park, Jeonghyeon Gu, Sangseon Lee, Sun Kim

**Affiliations:** 1 Department of Pathology and Laboratory Medicine, Weill Cornell Medicine, New York, NY 10065 USA; 2 Interdisciplinary Program in Bioinformatics, Seoul National University, Seoul, 08826 South Korea; 3 Department of Computer Science and Engineering, Seoul National University, Seoul, 08826 South Korea; 4 Interdisciplinary Program in Artificial Intelligence, Seoul National University, Seoul, 08826 South Korea; 5 AIGENDRUG Co., Ltd, Seoul, 08826 South Korea; 6 Institute of Computer Technology, Seoul National University, Seoul, 08826 South Korea

**Keywords:** Subcellular localization, Data mining, Cancer, Bioinformatics, Web system, Multi-omics, Protein abundance

## Abstract

**Background:**

Compartments such as subcellular organelles provide adequate environments and interacting partners to the constituent proteins so that their dedicated functions can be carried out properly. While mRNA or protein abundance data is commonly used in computational analysis, data mining from a single abundance data often do not provide sufficient information towards diseases like cancer that introduce various perturbations to biological functions. In addition, transcriptomic and proteomic data do not agree to a large degree. Such challenges make it imperative to comparatively analyze data of multiple omics in terms of subcellular locations to better understand the functional dynamics of cancer. In this paper, A Location-wise Proteome/transcriptome Abundance Comparative Analyzer (ALPACA) is introduced, a web-based visual data mining system for subcellular location-specific knowledge mining from multi-omics data.

**Results:**

ALPACA calculates location enrichment distributions from cancer abundance data and visually presents these in a pathway-specific manner, helping researchers observe biological function discrepancies. The effectiveness of ALPACA was demonstrated by reproducing known cancer-related genes and pathways across various types of cancers. For example, ALPACA was able to identify overexpression of TP53 and underexpression of DIABLO/Smac in basal-like breast cancer, as well as high discrepancy in Fc gamma R-mediated phagocytosis pathway among gynaecological cancers with an implicated gene GSN.

**Conclusions:**

ALPACA compartmentalizes the whole transcriptome and proteome abundance data subcellular location-wise and visually presents the differences of selected cancers using subcellular locations and biological pathways. The filtering and sorting functions of ALPACA add biological pathway context to the data analysis to help identify potential biomarkers. ALPACA is available at https://github.com/mwpak/ALPACA.

**Supplementary Information:**

The online version contains supplementary material available at https://doi.org/10.1186/s12920-026-02440-4.

## Background

Cancer is a disease of uncontrolled growth of abnormal cells, which is caused by changes in multi-omics features such as mutation rate, copy number aberration, and altered transcriptomic/proteomic expression. In particular, proteins are the primary machinery that carries out essential biological functions in subcellular location-specific ways. Thus, to investigate biological malfunctions, it is essential to analyze the dynamics of proteins by comparative analysis of multi-omics data.

### Motivation

#### Inconsistency between transcriptome and proteome abundance

Transcriptomic and proteomic abundance are commonly explored to study the phenotypic outcomes of cancers. However, the information derived from two different omics is often inconsistent [1–3]. Such inconsistency between the two omics poses more challenges in cancer research. Functional mechanisms of proteins such as subcellular localizations should further be considered to understand in which functional context does the inconsistency occurs and which potential biomarkers are associated with it.

#### Importance of spatial distributions of proteins at subcellular location level

Subcellular compartments, such as organelles inside a cell, accommodate function-specific environments for proteins they harbor [4]. In various diseases including cancer, the disease phenotypes are known to be associated with protein dysfunctionality and localization [5, 6]. Studies show that subcellular location-wise distributions of proteins vary widely among different types of diseases and conditions [6–8]. Therefore, due to the influence of subcellular localization to the functions of proteins, it is imperative to take protein localization into account when comparatively analyzing cancer dynamics and phenotypes. However, considering the various types or subtypes of cancer and the diversity of biological functions, it would be costly and time-consuming to experimentally analyze all the combinations. Therefore, a data mining system that computationally analyzes various types of cancers in the context of protein localizations and biological functions is much needed.

#### Existing work

There are many standalone analysis tools available for transcriptomic or proteomic abundance data. Each one provides a specific analysis functionality of its own, but only few of them are designed to mine knowledge from abundance data and visually present the result for intuitive perception of the information. Most existing tools specialize only in either abundance analysis or data visualization. Moreover, the tools that incorporate subcellular localization information are scarce. MSstatsTMT [9], TopPIC [10] and SDAMS [11] are command-line tools that detect differentially abundant proteins from mass spectrometry-based protein abundance data. While these tools enable researchers to explore abundance data, the way of visualizing the information is limited since the result is presented in command-line interface. Tools such as Heatmapper [12] and expVIP [13] visualize gene expression data through a web-based graphical user interface, but they are limited in providing further biological interpretation because they do not provide biological function-specific views.

More importantly, none of the tools mentioned above analyze data in the context of subcellular localization. Most of the available tools that utilize subcellular localization data are for protein localization prediction task [14–17]. These tools focus on predicting the location labels given the information of a protein such as amino acid sequence as input, rather than on data mining or data visualization. COMPARTMENTS does visualize subcellular localization information of proteins but only annotates a single protein at a time [18]. CellMap is a tool dedicated to visualizing protein in the context of subcellular localization as well as protein-protein interaction [19]. However, both COMPARTMENTS and CellMap focus only on annotating proteins in terms of localization and do not mine knowledge from proteomic/transcriptomic abundance data. To the best of our knowledge, there is no visual data mining system for performing biological function-specific analysis in subcellular location-wise fashion. Furthermore, none of the existing tools simultaneously compares multiple abundance data of different omics such as transcriptomic expression and proteomic quantification.

### Introduction to ALPACA

**Fig. 1:**
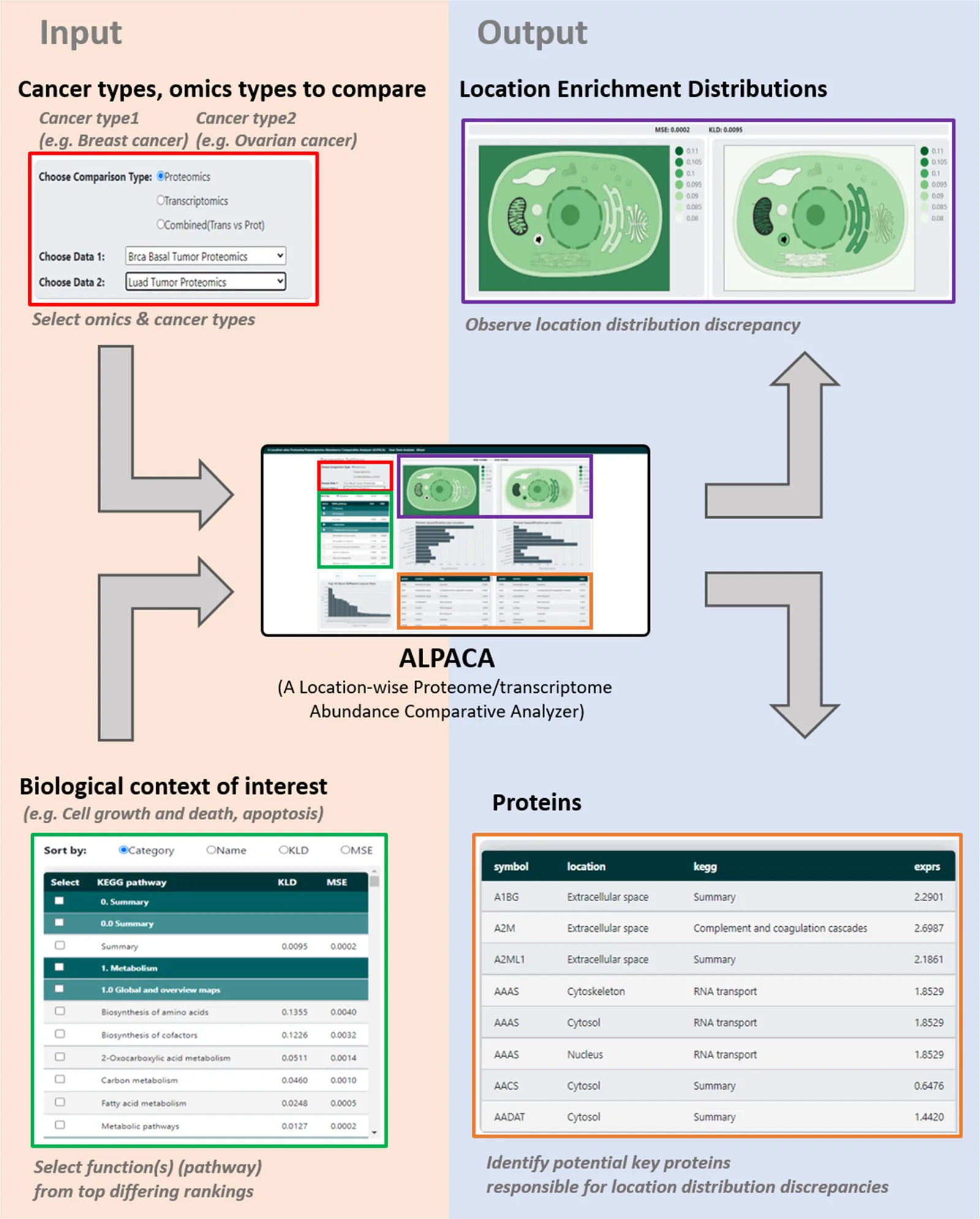
The overview and data mining process of Location-wise Protein Abundance Comparative Analyzer (ALPACA). (1) Users first need to choose a cancer type pair and omics for comparative analysis. (2) ALPACA can restrict the analysis to a biological function by filtering the abundance data to include only the proteins related to the KEGG pathway(s) of interest. (3) The location enrichment distributions are visually presented with cell heatmaps. The location enrichment is represented by the varying color intensities. (4) Users can click on a location to identify the proteins, which may contribute to the discrepancies caused in the selected location

In this paper, we propose a visual data mining system, ALPACA (A Location-wise Proteome/transcriptome Abundance Comparative Analyzer). Unlike the existing tools, ALPACA focuses on both location-wise abundance data analysis and data visualization. ALPACA is designed to enable researchers to explore abundance data in a simple and intuitive way and retrieve valuable information in terms of a biological context of interest. This way, researchers can efficiently compare data of different cancer and omics types with little effort. Since ALPACA compares location distributions in multiple levels of granularity from the whole transcriptome/proteome to individual proteins, researchers may identify in which locations various proteins are enriched differently in specific cancer conditions. The overview of the **general data mining steps** when using ALPACA are as follows (Fig. 1):

- **Step1.** Data Selection
  - Select the omics types and cancer types of interest to compare
- **Step2.** Pathway Selection
  - Select one or more pathways to add biological context to the analysis
  - Pathways can be sorted by the degree of discrepancies between the two cancers
  - Data is filtered to include only the proteins that are related to the selected pathway(s)
- **Step3.** Presentation of Location-specific Heatmaps
  - Observe location enrichment distribution discrepancies between the two cancers
- **Step4.** Identification of Potential Key Proteins
  - Examine proteins that may cause the similarity or dissimilarity of the location enrichments.

Detailed illustration of the data mining process is described in Data mining process for potential key protein identification section as well as the Case Study sections.

We demonstrate the utility of our system by identifying differentially enriched subcellular locations and their potential key actor proteins that differentiate phenotypes among different cancer types and subtypes.

## Implementation

### Data

#### Protein localization data

There are many resources that provide the localization information of proteins, including COMPARTMENTS [18], UniProt [20, 21], Human Protein Atlas [22, 23] and LocTree3 [24]. COMPARTMENTS is one of the most up-to-date and comprehensive databases, which provides evidence for each localization information via four evidence channels, namely, knowledge, experiments, text-mining and predictions. In this study, we parsed the positive records in the benchmark set of COMPARTMENTS which are localization labels supported with the highest level of confidence. We then unified the four data sources mentioned above using the 11 location labels of the COMPARTMENTS benchmark set as the canonical location labels and obtained 34,172 protein-location pairs in total. The 11 location labels and the numbers of constituent proteins are shown in Table 1. A protein can be localized to multiple subcellular compartments, hence a protein can have multiple location labels. However, there is no database that evidently specifies the localizations of proteins in condition-specific way. Therefore, in this study, the protein abundance is mapped to the locations where it is known to be localized.

**Table 1:** Location labels from COMPARTMENTS, UniProt, Human Protein Atlas and LocTree3 are unified to the 11 location labels of the COMPARTMENTS benchmark set

Location Labels	#Constituent Proteins
Nucleus	10040
Cytosol	9167
Plasma membrane	5234
Mitochondrion	2226
Cytoskeleton	1873
Golgi apparatus	1671
ER	1601
Extracellular space	1536
Endosome	442
Lysosome	263
Peroxisome	119

#### Proteomics and transcriptomics data

The transcriptomic expression and proteomic quantification data of various cancer types and subtypes were obtained from CPTAC Python data application programming interface (API) [25]. Both the transcriptomics and proteomics datasets were collected for breast cancer, clear cell renal carcinoma, colon cancer, lung adenocarcinoma, glioblastoma multiforme, endometrial cancer and ovarian cancer. For breast cancer, the transcriptome and proteome abundance data were collected at the subtype level as well. The included subtypes were from PAM50 subtype [26], namely Her2, Luminal A, Luminal B, Basal and Normal-like.

#### Biological pathway data

To effectively investigate the location-wise distribution discrepancies between different cancers, our system is capable of performing comparative analysis of location-wise distribution in terms of biological functions. The unit of biological function used in our system is the KEGG pathway [27]. Comparing location-wise distributions of different cancers in pathway-specific way would allow users to narrow down the scope of the analysis to the biological functions of interest. The KEGG pathway information used in this paper was obtained from the KEGG pathway database (https://www.genome.jp/kegg/pathway.html).

### A location-wise proteome/transcriptome Abundance Comparative Analyzer (ALPACA)

#### Location abundance enrichment

In order to calculate the distributions of protein expression or quantification over different locations, the abundance values of each protein are aggregated by taking an average value across all the samples for each cancer type. It is worth noting that for the proteomic quantification datasets, the values provided by the CPTAC API represent relative protein abundance. In particular, the abundance values are ratio values to a pooled reference and are then log transformed [25]. Directly calculating the means from the log transformed ratios would not be proportional to the means of the raw abundance values. Therefore, the dataset values from CPTAC API are exponentiated before taking the mean. Equation 1 illustrates the aggregation process.

1 $$\begin{aligned} Q_{p} = \sum \limits _{s \in S}\frac{\exp (q_{p,s})}{n} \end{aligned}$$

where $Q_{p}$ denotes the quantity value of a protein *p* which is either an aggregated transcriptomic expression value or protein abundance value. *S* denotes a set of all samples and *n* is the total number of samples. Based on the aggregated quantities of the proteins, the enrichment values of the 11 locations are calculated by the protein values belonging to each location as follows (Eq. 2):

2 $$\begin{aligned} \mathop {\mathbb {E}[Q_{p,\boldsymbol{\ell }}]} = \frac{\sum \nolimits _{p}Q_{q} \ \cdot \ \mathbb {1} (p \in \boldsymbol{\ell })}{|\{p\ |\ p \in \boldsymbol{\ell }\}|} \end{aligned}$$

where $\mathop {\mathbb {E}[Q_{p,\boldsymbol{\ell }}]}$ is a location enrichment value of the proteins that are known to localize to location $\boldsymbol{\ell }$. $\mathbb {1} (p \in \boldsymbol{\ell })$ is the indicator function that takes on the value 1 if the protein is known to localize to the location and takes on the value 0 otherwise. All enrichment scores of the locations are concatenated together to form a single vector $\tilde{\boldsymbol{\ell }}$. Finally, the distribution of the location enrichments over the 11 subcellular locations $D(\boldsymbol{L})$ is calculated by feeding the vector $\tilde{\ell }$ into the softmax function [28] to obtain the normalized distributions of protein quantity over subcellular locations as illustrated in Eq. 3:

3 $$\begin{aligned} D(\boldsymbol{L})^{\boldsymbol{\ell }} = \frac{\exp (\mathbb {E}[Q_{p,\boldsymbol{\ell }}])}{\sum \nolimits _{\boldsymbol{\ell }}^{\boldsymbol{L}} \exp (\mathbb {E}[Q_{p,\boldsymbol{\ell }}])} \end{aligned}$$

where $\boldsymbol{L}$ denotes the set of all 11 locations.

#### Quantitative difference metrics of location distributions

Having computed the location enrichment distributions, users can explore various combinations of cancer pairs and pathways of interest. ALPACA visually presents protein abundance concentrations distributed across the subcellular locations using colored cell heatmaps and tables that show the abundance values of the constituent proteins. This way, the differentially enriched locations are perceptually visible, and the proteins related to phenotypic differences can be captured by observing their abundance values presented in the table. However, just having the distribution values is not so useful without a way to effectively search through various combinations of cancers and pathways. Users may not know which pathways to investigate beforehand. In many cases, we suspect that researchers would want to find which pathways have high or low protein abundance perturbations between the cancer conditions of interest. The primary way of guidance provided by ALPACA is through quantifying the measure of discrepancies between a cancer pair of interest in terms of KEGG pathways. By sorting the pathways according to the degree of difference between the selected cancers, researchers can easily get a sense of which biological context the two cancers are most different or similar at. The difference metrics used to compare the distributions of the locations are Mean Squared Error (MSE) and Kullback-Leibler Divergence (KLD) [29] (Eqs. 4 and 5).

4 $$\begin{aligned} MSE(D_{c1}(\boldsymbol{L}), \ D_{c2}(\boldsymbol{L})) = \frac{1}{|\boldsymbol{L}|}\sum \limits _{\boldsymbol{\ell } \in \boldsymbol{L}}(D_{c1}(\boldsymbol{L})^{\boldsymbol{\ell }} - D_{c2}(\boldsymbol{L})^{\boldsymbol{\ell }})^{2} \end{aligned}$$

5 $$\begin{aligned} KLD(D_{c1}(\boldsymbol{L}) \ || \ D_{c2}(\boldsymbol{L})) = \sum \limits _{\boldsymbol{\ell } \in \boldsymbol{L}}D_{c1}(\boldsymbol{L})^{\boldsymbol{\ell }}log(\frac{D_{c1}(\boldsymbol{L})^{\boldsymbol{\ell }}}{D_{c2}(\boldsymbol{L})^{\boldsymbol{\ell }}}) \end{aligned}$$

where the cancer types being compared are denoted by *c*1 and *c*2.

Quantitatively representing the difference between the two cancers in terms of a biological pathway would guide users to narrow down on the important pathways and proteins to investigate.

#### Data mining process for potential key protein identification

Depending on the purpose of the data mining analysis, ALPACA can draw different kinds of information from the data. Among the various use cases of our data mining system, the most common use would be to help identify potential biomarkers of a cancer. Specifically, upon selecting a desired pair of cancer types, our system presents a location-specific heatmap representing the corresponding protein abundance by color intensity for each of the selected cancers (Fig. 1 (1)). Our system also presents the complete list of KEGG pathways that can be sorted by name or a difference metric that quantitatively represents the discrepancy between the two selected cancers in the viewpoint of each pathway (Fig. 1 (2)). The displayed biological pathways are organized in hierarchical categories as presented in the KEGG database. This makes it easy for users to spot pathways related to a biological aspect of interest. Our system also allows users to select multiple pathways to view the comparison of the cancer pair in a broader perspective. When the user selects one or more pathways, our system parses the data to include only the proteins that comprise the selected pathways and recomputes the location distribution values. Once the updated pathway-specific location heatmaps are rendered, the degrees of how different each location is between the different cancers would be perceptible through the varying color intensities updated accordingly (Fig. 1 (3)). Users can then simply click on a location that discriminates the two cancers to view the proteins localized to the location (Fig. 1 (4)). This way, proteins that exert the most influence on the location discrepancy between the two cancers can be identified.

#### System architecture

**Fig. 2:**
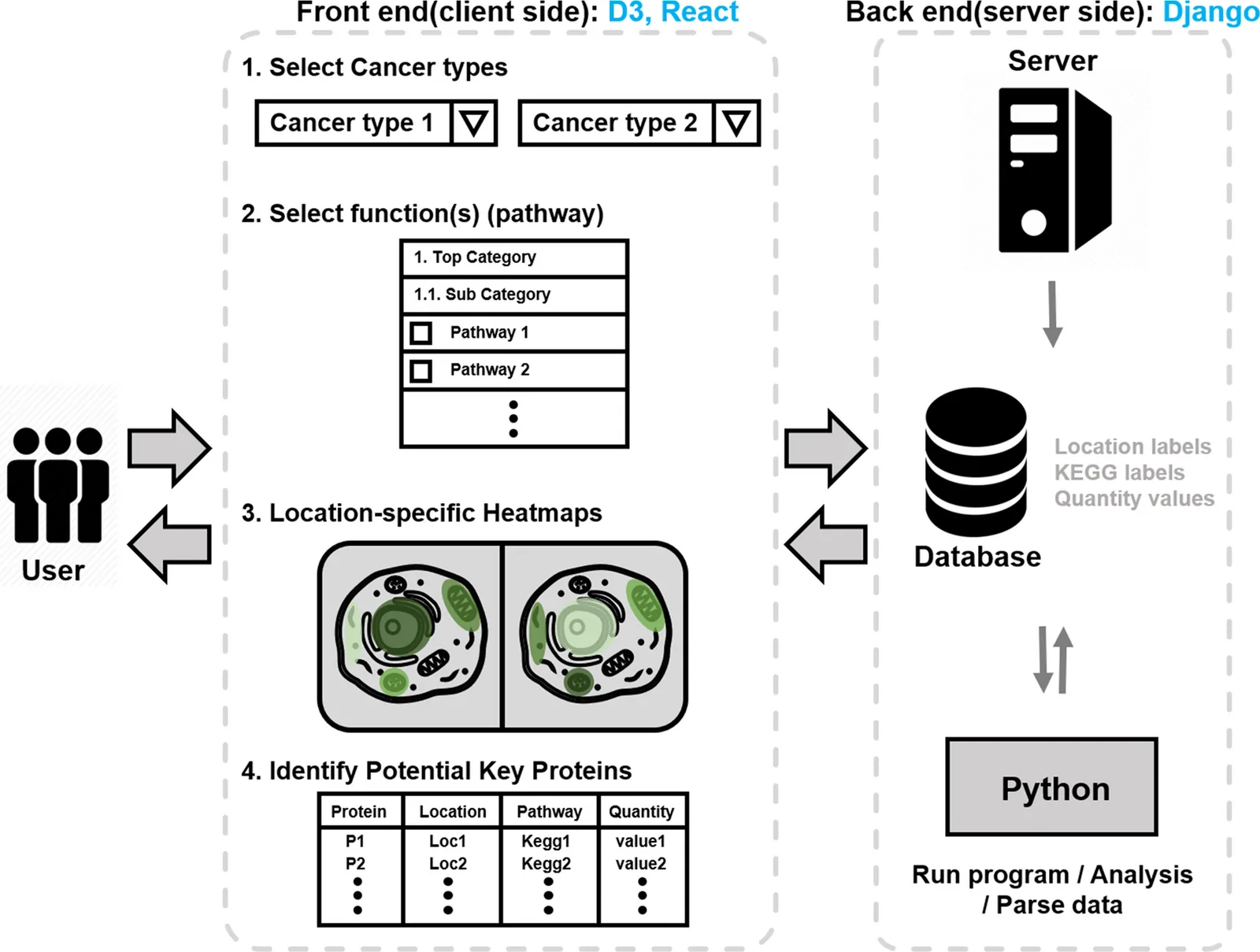
ALPACA system structure. Users interact with the frontend of the system in unidirectional data flow. The interactions such as choosing a cancer type or a KEGG pathway triggers the backend to fetch and parse the data and to re-render the frontend

Figure 2 shows the overview of the system architecture. Our system utilizes a Python backend framework called Django which serves the server and stores data. The frontend visualization is handled by D3 and React Javascript library.

## Results

### Demonstration of the power of location information use in disease gene detection analysis

We demonstrate the power of protein localization information through disease gene detection analysis. Disease-associated genes are detected for each cancer type by statistical testing and location information-based filtering. The detected genes are then compared with known disease genes. Known disease genes of the cancers are collected from DisGeNET  [30]. Using the disease genes as the ground truth genes, Differentially Expressed Gene/Protein (DEG/DEP) analyses are performed on transcriptomic expression and proteomic quantification data respectively to see where in the rank the ground truth genes are distributed.

The Welch’s t-test with Bonferroni multiple testing correction is used for the differential expression analysis for the both omics data [31, 32]. The rankings of the genes or proteins are obtained according to the adjusted *p*-value of each gene and the genes with adjusted *p*-value lower than 0.05 are considered as DEGs/DEPs. The location list with the most significant discrepancies between normal and tumor conditions is then obtained by calculating average abundance differences for each of the 11 locations. Finally, the DEGs/DEPs ranked lists are obtained by keeping only proteins that belong to the most discriminative locations.

To demonstrate the result, we first compared the rankings of the ground truth genes with and without the location filtering. For the case of lung adenocarcinoma (LUAD) proteome as an example, by applying the location filtering at the threshold of the top 1 most discriminative location, 6 ground truth genes are detected with the ranking of the last ground truth gene, MET, of 297 (Table 2). On the contrary, 44 ground truth genes are detected without location filtering, but the sixth ground truth gene has the rank of 1,394. The same last ground truth gene, MET, in particular has the rank of 4,395. This result shows that without location filtering, up to 1,400 DEPs need to be investigated to include ground truth genes while with location filtering, much smaller number of genes, 300 genes, are needed to be investigated. Despite the fewer number of ground truth genes detected, the removal of false positives and the rank-boosting effect of the location filtering are far more significant.

**Table 2:** Ranks of the ground truth genes with and without location filtering

Without Location Filter		With Location Filter	
Symbol	Rank	Symbol	Rank
PIK3CA	312	FGFR1	94
APC	367	AXL	117
PXN	535	KDR	205
LRRC59	736	LGALS1	231
FOXO3	1261	ADAMTS1	238
ERGIC3	1394	MET	297
...	...	...	...
KRAS	4373		
MET	4395		

**Fig. 3:**
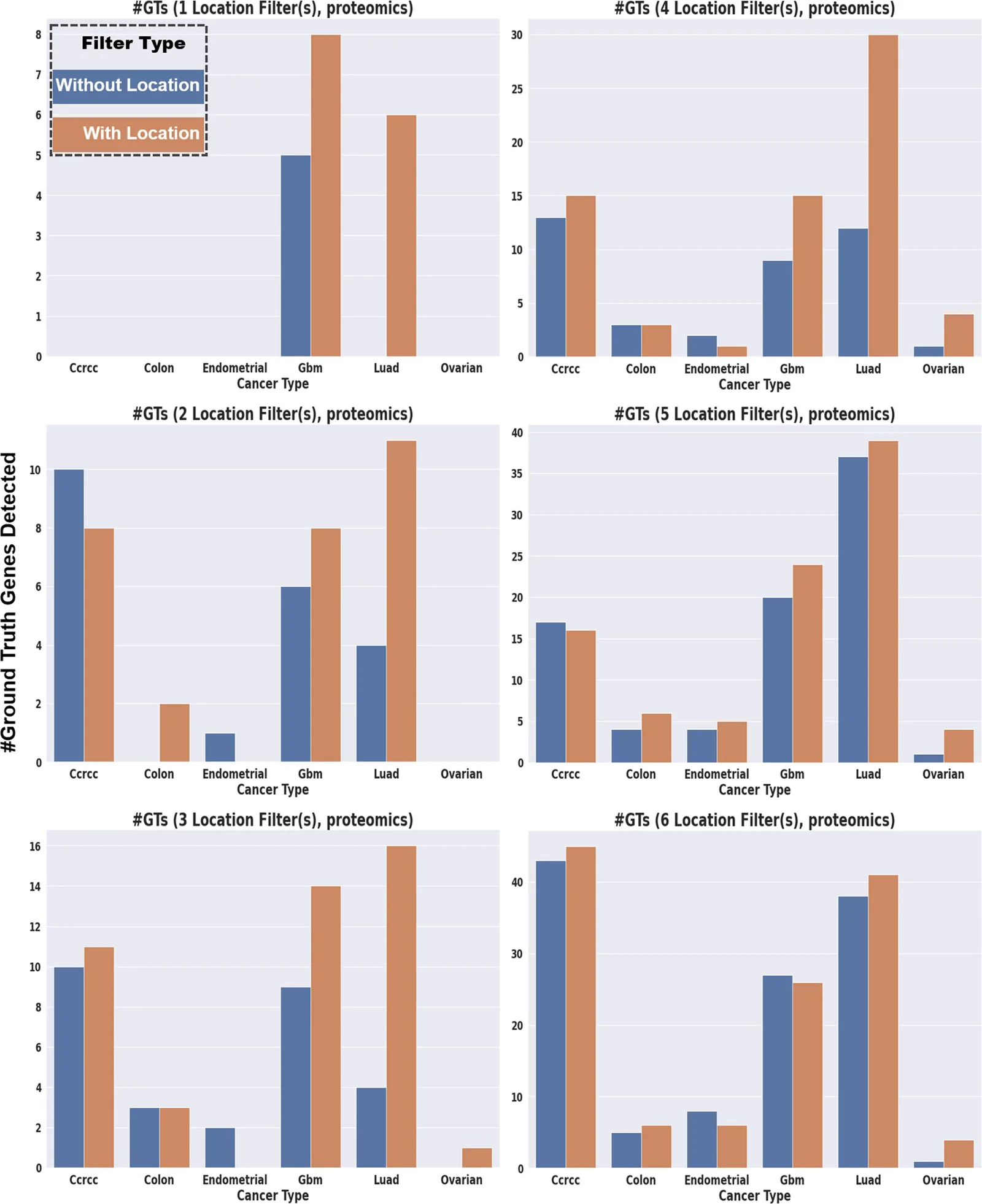
The number of ground truth genes with and without location filtering. Orange bars represent the number of ground truth genes while applying the location filtering. Blue bars represent the number of ground truth genes filtered using only the *p*-values to obtain the same number of DEPs as the location filtering case. Each plot shows a different level of location filtering threshold. In all cases, more than four out of six cancer types show higher number of detected ground truth genes with location filtering than without location filtering

Similar results were obtained with different threshold numbers of top differing locations. Such result shows that adding location information can reduce false positive genes situated in between the ground truth genes in the ranking list and boost the ranks of the ground truth genes so that more significant genes can be identified. To generalize the results further, we compared the number of ground truth genes detected with and without the location filtering (Fig. 3). The location filters were applied while varying the thresholds of the number of top differing locations to avoid restricting the result to a particular case. For a fair and quantitative comparison, the number of top DEPs without the location filtering is matched with the number of DEPs with the location filtering based on the *p*-values. Except for a few cases of endometrial (EDM) cancer and clear cell renal cell carcinoma (Ccrcc) cancer, the number of captured ground truth genes is greater with location filtering applied. Although the pattern is not as distinct in the transcriptomics data where the number of ground truth genes does not differ much between the two conditions, similar patterns can also be found in more than half of the cases. The results for the transcriptome can be found in the Additional file 1.

### Data mining case Study1 (breast cancer subtypes)

We demonstrate the usefulness of ALPACA by comparatively analyzing different combinations of cancer types utilizing the system guidance under different use case scenarios.

#### Rediscovery of biomarkers for discriminating cancer subtypes

**Fig. 4:**
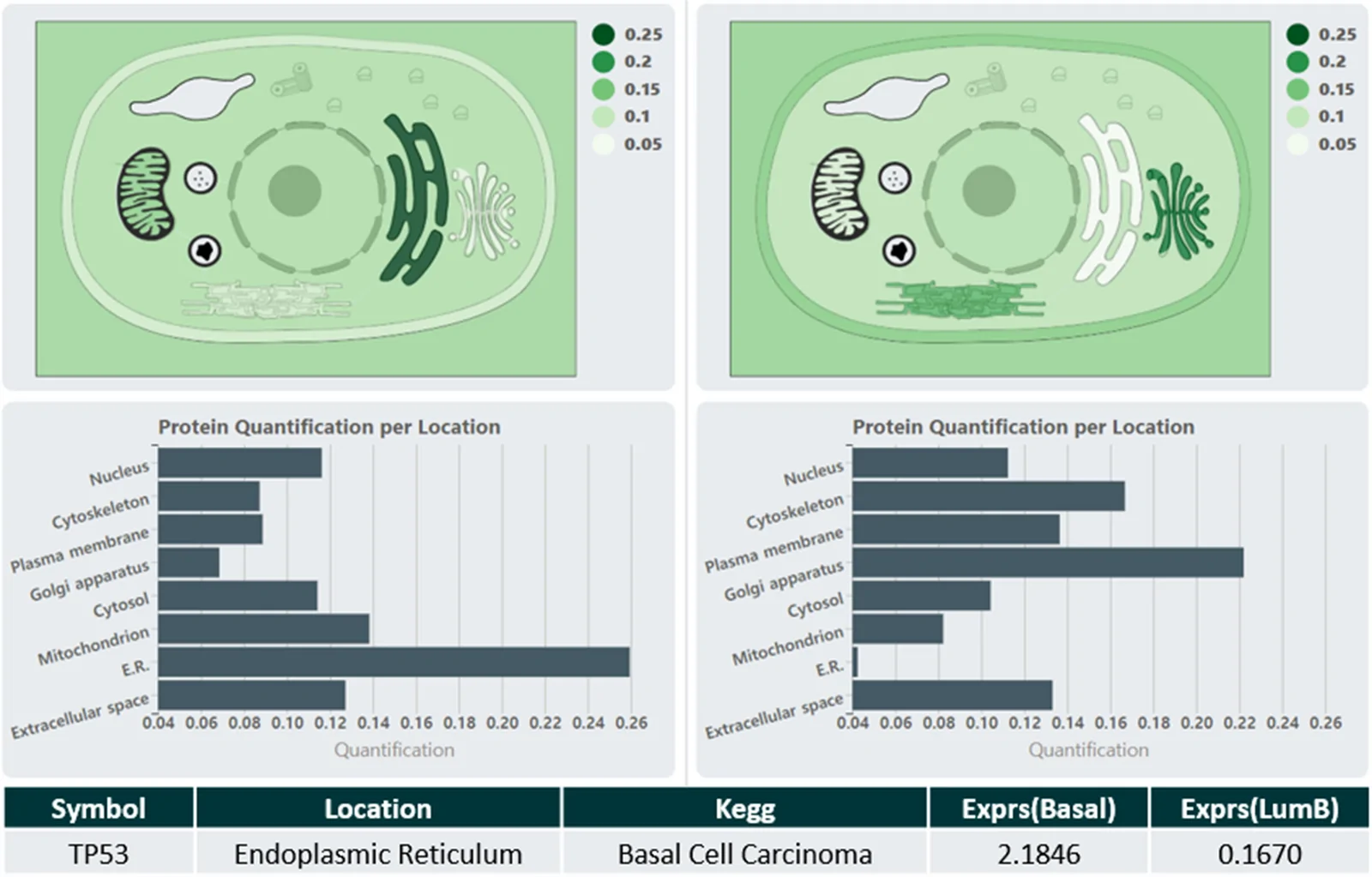
Basal (left) vs LumB (right) Proteomics, Pathway: Basal Cell Carcinoma, Location: ER. A large gap of location enrichment distributions between the two cancers is observed in the ER. The discrepancy is due to the abundance difference of TP53

Basal breast cancers are known to have the worst prognosis of all subtypes of breast cancer [33]. We manually curated the pathways that are related to cancer, and selected those with high KLD values. Using ALPACA, the locations that exhibit significant difference in their distribution were further selected. Figure 4 and Additional file 2 show some of the location-specific heatmaps that reveal the discrepancies observed between Basal and Luminal subtypes and different omics data in terms of various pathways. Basal cell carcinoma is a pathway related to cancer in epithelial skin cells. According to the cell diagram panels of our system, the pathway exhibits a large gap of endoplasmic recticulum (ER) in proteomics between basal and both luminal breast cancers (Fig. 4). This gap is explained by the overexpression of *TP53* gene in the basal subtype. *TP53* is a tumor suppressor gene that encodes *P53*. *P53* regulates apoptosis when DNA is damaged, thus replacing the damaged cells with ones with healthy copy of DNA. Therefore, *TP53* is normally expressed in nucleus and exported to cytoplasm when DNA is damaged [34, 35]. The overexpression of *TP53* also implies its mutations [36]. Furthermore, overexpression of *TP53* is reported to be a clear sign of poor prognosis in breast cancer [37–39] and also other cancers [40–42], which is consistent with findings from our knowledge mining.

Similarly, we found that SLC11A2 and TFRC from ferroptosis pathway in lysosome significantly overexpressed in basal breast cancer proteomics compared to luminal ones (see Additional file 2 (a)). In transcriptomics data, *TFRC* from ferroptosis in extracellular space was significantly overexpressed for basal (see Additional file 2 (b)). TFRC which is found in both the proteomics and the transcriptomics data is a transferrin receptor protein relevant for cellular uptake of iron [43]. We can hypothesize from these findings that overexpression of TFRC both in lysosome and in extracellular space may be a reason of poor prognosis of basal breast cancers. Literatures such as [44] support the importance of ferroptosis in a sense that basal breast cancers are more susceptible to ferroptosis and thus can be used to targeted therapy [45].

#### Data mining based on prior knowledge

We investigated the discrepancies between basal-like and HER2-positive breast cancers with ALPACA. The case study was conducted under the scenario that the user utilizes prior knowledge that apoptosis is significantly related with basal-like and HER2 breast cancer subtypes [46]. Apoptosis is a genetically programmed process of eliminating damaged or redundant cells by activation of caspases. In our system, this pathway shows significant difference in mitochondria, which is partly due to DIABLO/Smac (see Additional file 3 (a)). DIABLO/Smac is a mitochondrial protein that promotes cytochrome c-dependent caspase activation [47]. A recent study suggested that DIABLO/Smac is one of HER2-specific genes that showed some correlation between copy number and gene expression profiles [48]. Furthermore, basal-like breast cancers are known to be highly proliferative and anti-apoptotic, which can also be explained by low expression level of DIABLO/Smac that participates in apoptosis pathway [46].

We also looked into another pathway well known for its implication in cancer, P53 signaling pathway. *P53* is a tumor suppressor gene, and has its role in conserving stability by preventing genome mutation [49], hence called “the Guardian of the Genome”. *P53* can act as a transcription factor that affects cell cycle arrest or cellular senescence [50]. According to  [51], basal-like breast cancers have a few markers associated with poor survival, including a gene called *PTEN*, which usually shows low expression in this subtype. This marker is consistent with the finding of our system which was able to detect that *PTEN* showed lower expression in basal-like breast cancers compared to HER2-positive subtype (see Additional file 3 (b), (c)).

### Data mining case Study2 (gynaecological cancers)

#### Data mining guided by difference metrics

**Fig. 5:**
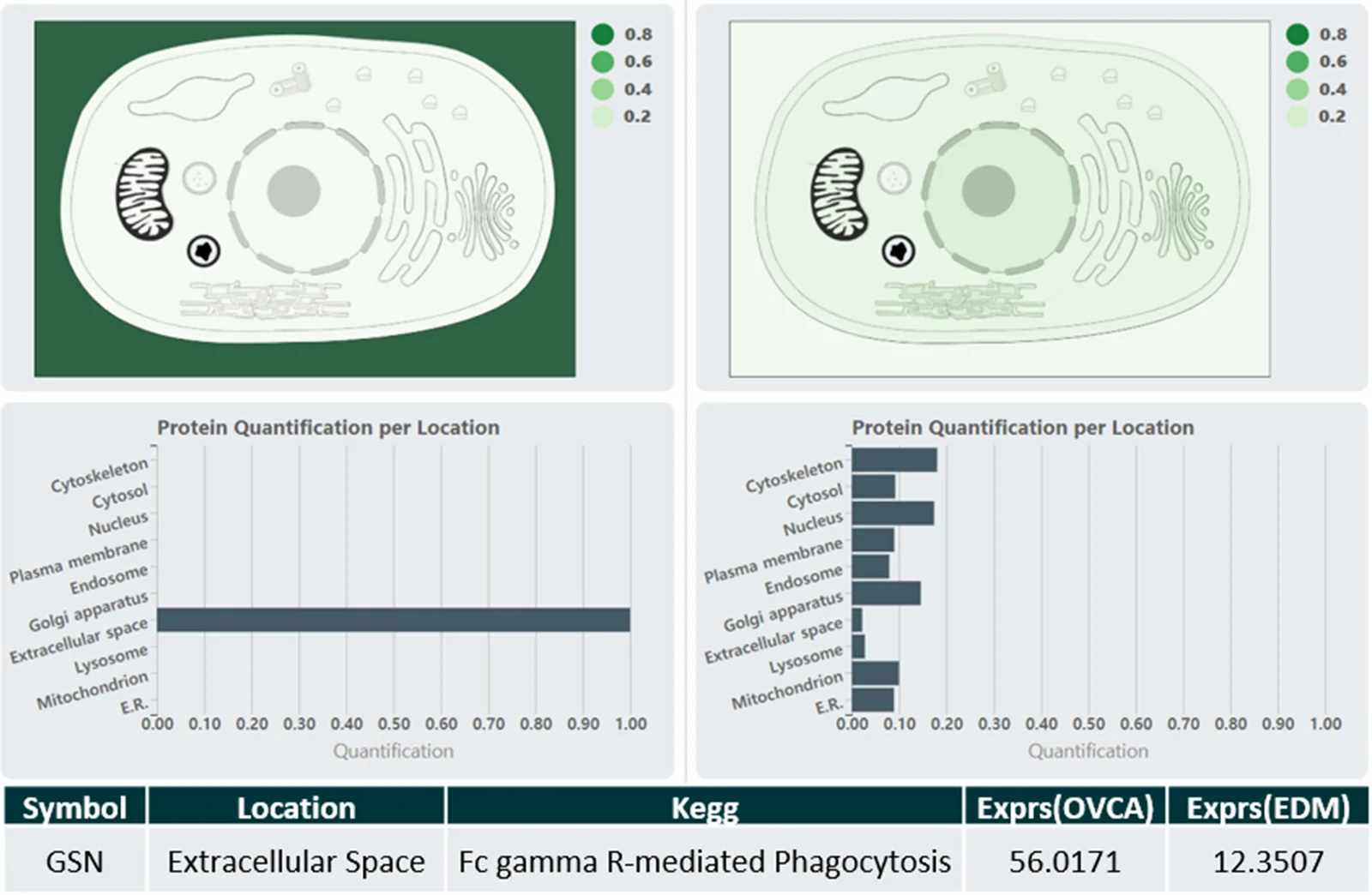
OVCA (left) vs EDM (right) Transcriptomics, Pathway:Fc gammaR, Location: Extracellular space. A large gap of location enrichment distributions between the two cancers is observed in extracellular space. The discrepancy is due to the abundance difference of *GSN*

In this case study, we allowed ALPACA to guide identification of potential biomarkers relevant to the phenotypes of the gynaecological cancers, namely endometrial cancer (EDM) and ovarian cancer (OVCA) without assumption based on prior knowledge about specific cancers. Sorting pathways by KLD scores, ALPACA immediately showed that the most distant pathway for transcriptomics data between EDM and OVCA is Fc gamma R-mediated phagocytosis. A distinct difference can be observed in extracellular space (Fig. 5). The gene that explains best for the corresponding discrepancy was gelsolin (*GSN*). GSN is an actin filament regulator protein known to promote cell motility, which is highly correlated with cancer progression and tumor cell migration [52]. Studies have also reported overexpression of *GSN* and its association with more aggressive phenotypes and worse prognosis in some cancers including gynaecological cancers [53–56]. In contrast, *GSN* is also known as a tumor suppressor and is downregulated in some cancers such as breast cancer [57, 58]. This agrees with the results shown by ALPACA and can easily be confirmed that the transcriptomic expression level of *GSN* is -0.05 in Breast cancer while it is 56.02 and 12.35 in OVCA and EDM, respectively, indicating that the expression of *GSN* is the highest in OVCA (Fig. 5, Additional file 4 (a)). Moreover, *GSN* is known to localize to multiple locations including cytoplasm and plasma [59]. One relatively well-studied isoform pGSN, which is localized to the plasma, is implicated in promoting OVCA cell survival through both autocrine and paracrine mechanisms thereby contributing to making OVCA the most lethal gynaecological cancer [56]. This result is also discovered by ALPACA since extracellular space discrepancy caused by *GSN* is more apparent than other localizations of *GSN* (see Additional file 4 (a), (b)).

#### Rediscovery of biomarkers for discriminating cancer types

**Fig. 6:**
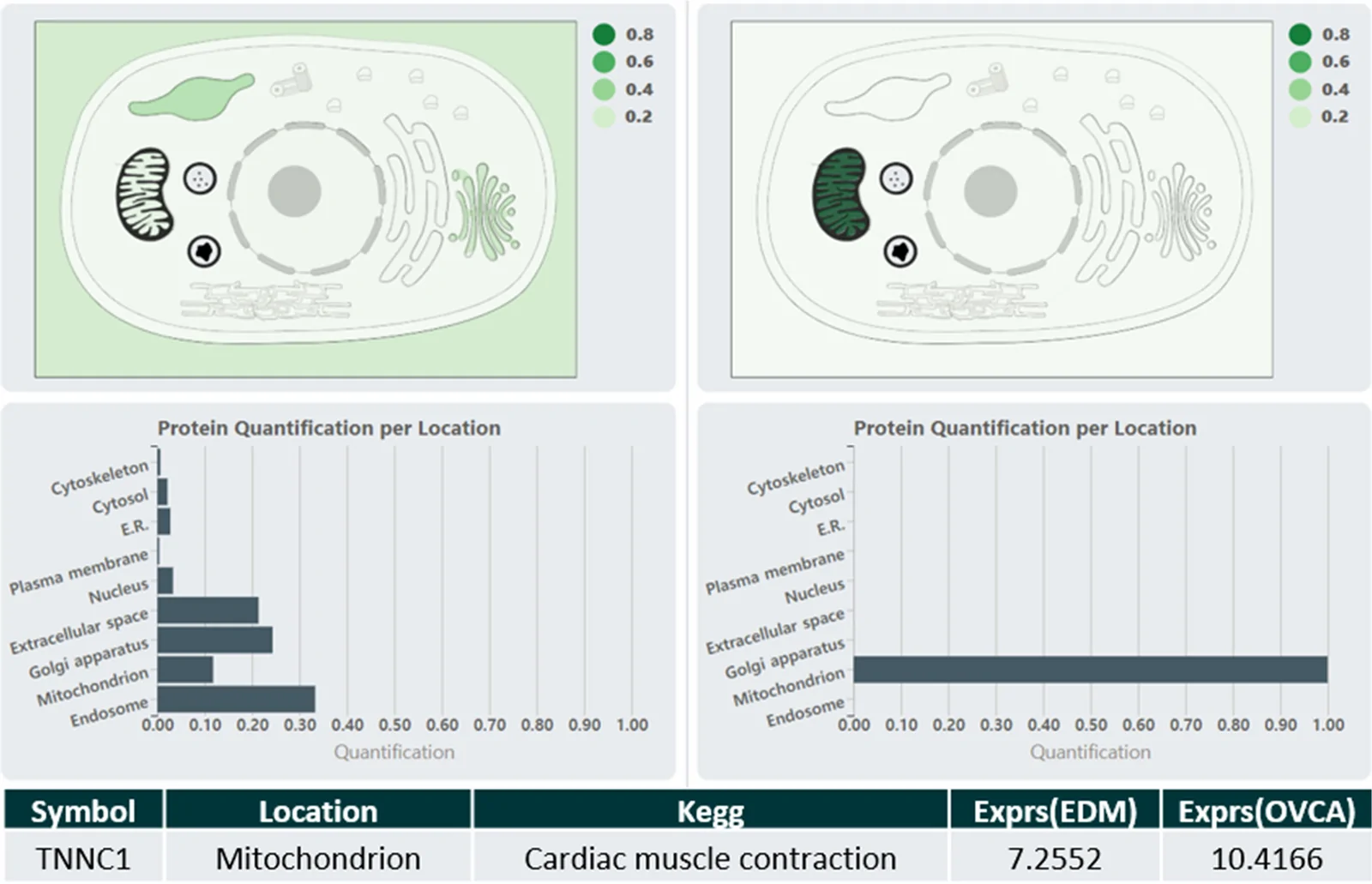
EDM (left) vs OVCA (right) Transcriptomics, Pathway: Cardiac muscle contraction, Location: Mitochondrion. A large gap of location enrichment distributions between the two cancers is observed in the mitochondrion. The discrepancy is partly due to the abundance difference of TNNC1

Another pathway high in KLD was cardiac muscle contraction. Using ALPACA, we were able to detect a potential biomarker with regard to the cardiac function in OVCA. Ovarian cancer is known to induce dysfunction in cardiac muscle and cardiac cachexia [60]. From the transcriptomic data of OVCA, the most distinct characteristic in location distribution was the enrichment of mitochondrion (Fig. 6). One of the contributing factors in the enrichment was a protein called TNNC1. TNNC1 is a skeletal/cardiac isoform of TnC which is a subunit of Troponin. The role of Troponin is to regulate striated muscle contraction [61]. In recent studies, TNNC1 is reported to be overexpressed especially in OVCA [62]. In addition, TNNC1 knockout is reported to result in decreased invasive phenotypes, proliferation and colony formation of cancer cells [63]. This agrees with the information of the transcriptomic data retrieved by ALPACA. As shown in Fig. 6, the gene expression value of TNNC1 in EDM is 7.2552 while the expression value is 10.4166 in OVCA. In proteomics abundance data, TNNC1 is only measured in OVCA while there is no measured value for TNNC1 in EDM (see Additional file 4 (c)). Based on the result from ALPACA and known correlation of TNNC1 overexpression to the behavioral change in EMT and actin microfilaments [63], we can hypothesize that the function of TNNC1 in mitochondrion may be implicated in this phenotype of OVCA.

## Conclusions

Cancer dynamics is highly complex that data from a single layer of omics does not give the complete information to study phenotypic characteristics of a cancer type. While transcriptomic and proteomic abundance carry crucial information of cell states, studies have found that the two omics often do not agree with each other. To better capture the biological context of interest, it is imperative to comparatively analyze them in the context of protein localization and biological pathway. In this paper, we present ALPACA, a visual data mining system that enables researchers to effectively compare multiple combinations of different cancer types, biological pathways and subcellular locations. With the metrics that quantitatively represent discrepancies of location enrichment distributions between cancers, our system guides researchers to determine important pathways or proteins that may be responsible for the discrepancy.

Using ALPACA, we demonstrated data mining case studies in various scenarios. As a result, we were able to rediscover known biomarkers of different cancer types guided by the system. We were also able to find not only consensus but also discordance between different omics and identified exactly which proteins were implicated in each case. There were cases where different omics data showed discordant results. For instance, GSN transcriptomic abundance, in OVCA from Case Study 2, was the highest compared to EDM and BRCA which is consistent with the literature. However, in proteomics data, the abundance of GSN in BRCA was the highest. The enrichment levels in the extracellular space of BRCA and OVCA were also reversed (see Additional file 4 (b)). This is an open research question of why the abundance of GSN in BRCA does not agree at the two different omics levels. We plan to keep building upon ALPACA for more comprehensive knowledge mining and visualization of the abundance and subcellular localization data. The source code for ALPACA can also be found ta https://github.com/mwpak/ALPACA. For stable availability, the source code includes the frontend part of the software where the data are fetched via permanent links. ALPACA is expected to provide researchers information-guided analysis functionality that leads to higher level comprehension of subcellular component-specific protein activity in cancer.

## Availability and requirements

 

- **Project name:** A Location-wise Proteome/transcriptome Abundance Comparative Analyzer (ALPACA)
- **Project home page:** https://github.com/mwpak/ALPACA
- **Operating system(s):** Platform independent
- **Programming language:** Python, JavaScript, HTML, CSS
- **Other requirements:** Django 3.2.4, Django-crispy-forms 1.12.0, Django Restframework 3.12.4, D3 7.0.0
- **License:** Not applicable
- **Any restrictions to use by non-academics:** Not applicable

## Supplementary Information

Additional file 1: Number of ground truth gene comparison bar charts (transcriptomics).

Additional file 2: Case study 1, Basal subtype Luminal subtype.

Additional file 3: Case study 1, Basal subtype Her2 subtype.

Additional file 4: Case study 2, Gynaecological cancers.

## Data Availability

All the datasets for this study are included in the manuscript and the Additional Files. The original proteomic and transcriptomic data are available on CPTAC python application programming interface (https://github.com/PayneLab/cptac). The original biological pathway data are available on KEGG pathway database (https://www.genome.jp/kegg/pathway.html), The specific urls for the pathways, genes and pathway-gene link information is available through the urls http://rest.kegg.jp/list/pathway/hsa, http://rest.kegg.jp/list/hsa, and http://rest.kegg.jp/link/pathway/hsa respectively. The original subcellular localization datasets are accessible at COMPARTMENTS (https://compartments.jensenlab.org/), UniProt (https://www.uniprot.org/), Human Protein Atlas (https://www.proteinatlas.org/about/download), and LocTree3 (https://rostlab.org/services/loctree3/downloads).

## Abbreviations

ALPACA: A Location-wise Proteome/transcriptome AbundanceComparative Analyzer
EMT: Epithelial Mesenchymal Transition
CPTAC: Clinical Proteomic Tumor Analysis Consortium
API: Application Programming Interface
ER: Endoplasmic Reticulum
EDM: Endometrial Cancer
LUAD: Lung Adenocarcinoma
OVCA: Ovarian Cancer
BRCA: Breast Cancer
LumA, LumB: Luminal A, Luminal B
